# Ginsenoside Rg1 as an Effective Regulator of Mesenchymal Stem Cells

**DOI:** 10.3389/fphar.2019.01565

**Published:** 2020-01-23

**Authors:** Fang He, Changyin Yu, Tao Liu, Huilin Jia

**Affiliations:** ^1^ Key Laboratory of Cell Engineering of Guizhou Province, The Affiliated Hospital of Zunyi Medical University, Zunyi, China; ^2^ Department of Oral and Maxillofacial Surgery, University Hospital of Tübingen, Tübingen, Germany; ^3^ Department of Neurology, The Affiliated Hospital of Zunyi Medical University, Zunyi, China; ^4^ School of Stomatology, Xi’an Medical University, Xi’an, China

**Keywords:** ginsenoside Rg1, mesenchymal stem cells, proliferation, differentiation, apoptosis, senescence, preclinical study

## Abstract

Recently, breakthroughs have been made in the use of mesenchymal stem cells (MSCs) to treat various diseases. Several stem cell types have been authorized as drugs by the European Medicines Agency and the U.S. Food and Drug Administration. The Chinese official document “Notification of the management of stem cell clinical research (trial)” was also published in August 2015. Currently, China has approved 106 official stem cell clinical research filing agencies and 62 clinical research projects, which are mostly focused on MSC therapy. Hence, the optimization and development of stem cell drugs is imperative. During this process, maximizing MSC expansion, minimizing cell loss during MSC transplantation, improving the homing rate, precisely regulating the differentiation of MSCs, and reducing MSC senescence and apoptosis are major issues in MSC preclinical research. Similar to artemisinin extracted from the stems and leaves of *Artemisia annua*, ginsenoside Rg1 (Rg1) is purified from the root or stem of ginseng. In the human body, Rg1 regulates organ function, which is inseparable from its regulation of adult stem cells. Rg1 treatment may effectively regulate the proliferation, differentiation, senescence, and apoptosis of MSCs in different microenvironments *in vitro* or *in vivo*. In this review, we discuss recent advances in understanding the effect of Rg1 on MSCs and describe the issues that must be addressed and prospects regarding Rg1 regulation of MSCs in preclinical or clinical studies.

## Introduction

Mesenchymal stem cells (MSCs) are a class of multipotent adult stem cells that show both self-renewal and high plasticity (Pittenger et al., 1999; He et al., 2018a; Wang et al., 2019). These cells can differentiate into adipocytes, osteoblasts, and chondrocytes *in vitro* and *in vivo* and secrete various anti-inflammatory cytokines and exosomes in different microenvironments (Chamberlain et al., 2007; Phinney and Pittenger, 2017). MSCs can be derived from many connective tissues and organ stroma, including bone marrow, Wharton's jelly of the umbilical cord, umbilical cord blood, adipose tissue, dental pulp, and periodontal tissues (Alison et al., 2000; Mastrolia et al., 2019). Meanwhile, these cells exhibit a fibroblastic morphology, adhere to a plastic surface when cultured *in vitro*, and share a common immunophenotype consisting of positive CD105, CD73, and CD90 expression and negative CD45, CD34, CD14, CD19, and HLA-DR expression (Wagers and Weissman, 2004). MSCs showing low immunogenicity are used for xenotransplantation to achieve immunomodulation and improve tissue regeneration (Jiang et al., 2002; He et al., 2018b). Thus, these cells are considered promising candidates in the management of several conditions, such as bone regeneration (Bruder et al., 1998), certain neurodegenerative disorders (Crigler et al., 2006), and graft-versus-host disease (Le Blanc et al., 2004).

**Figure 1:**
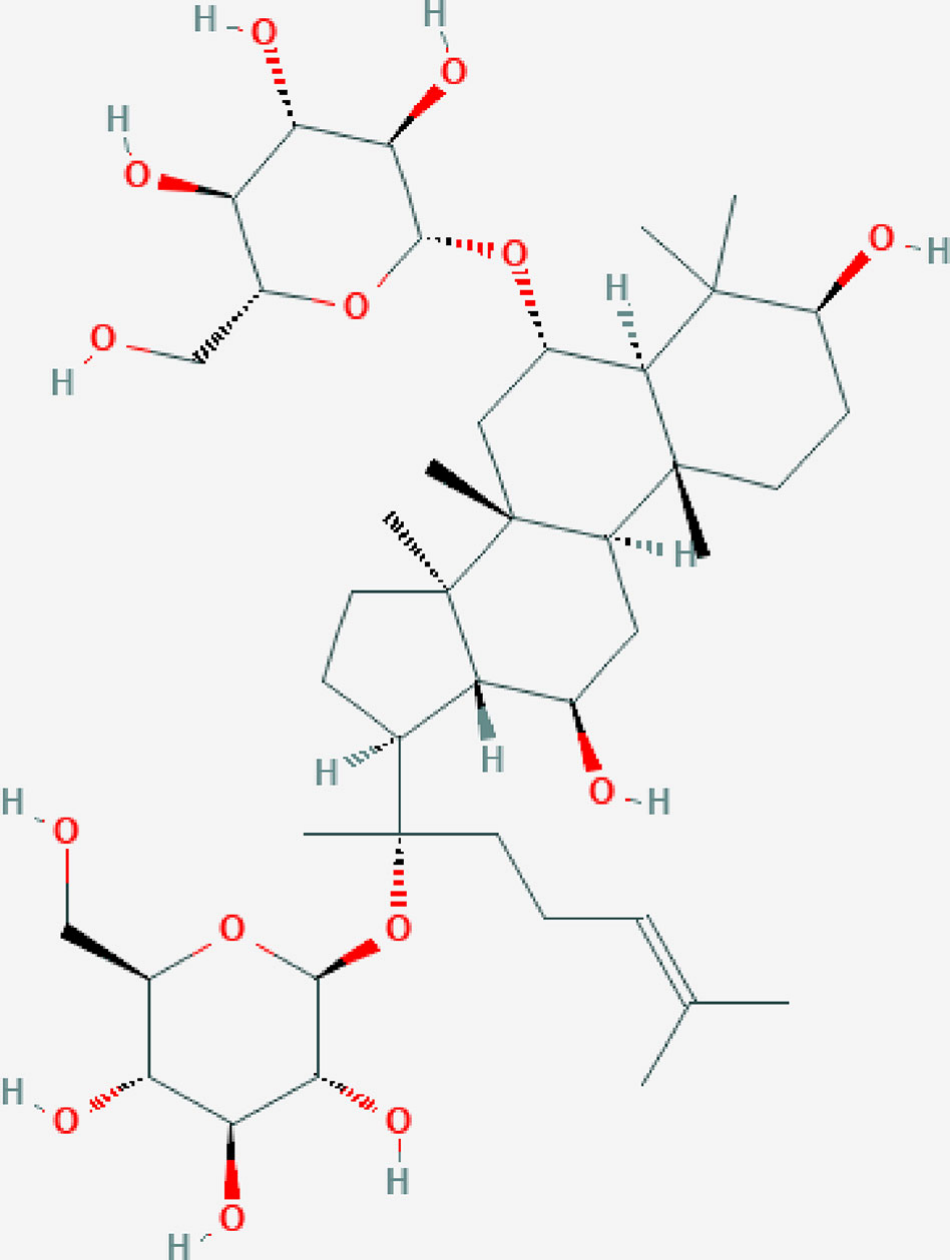
The molecular structure of ginsenoside Rg1.

Ginsenoside Rg1 (Rg1, molecular formula: C_42_H_72_O_14_, **Figure 1**, image from PubChem), which is derived from a hydride of a dammarane, is a monomer of a tetracyclic triterpenoid derivative. This molecule is mainly extracted and purified from the root or stem of ginseng (Leung and Wong, 2010; Xu et al., 2012). Ginsenosides are classified into 20(S)-protopanaxadiol (PPD) and 20(S)-protopanaxatriol (PPT) according to the hydroxylation position on their core triterpene saponin structure. Unlike PPD-type ginsenosides (e.g., ginsenoside Rb1 with four sugars) that are slowly excreted into bile, Rg1 with two sugars belonging to the PPT-type ginsenosides is primarily eliminated *via* rapid hepatobiliary excretion. Therefore, the specific molecular structure of Rg1 is a major determinant of Rg1 plasma pharmacokinetics and may also be a factor in drug interactions between Rg1 and its target molecules. In general, Rg1 can affect the nervous, cardiovascular, blood, and immune systems, showing various pharmacological activities (Lee et al., 1997; Fang and Limei, 2016). Rg1 has nutritional and protective effects on neurons and can reduce the apoptosis of nerve cells (Radad et al., 2004). Rg1 can be used to treat myocardial ischemia, long QT syndrome, and atherosclerosis by dilating coronary vessels, promote K+ outflow, and inhibit the proliferation of vascular smooth muscle cells (Wei et al., 2007; Lee and Kim, 2014). The effect of Rg1 on the endocrine system is similar to that of steroid hormones; for instance, Rg1 can compete with dexamethasone to bind glucocorticoid receptors to promote the secretion function of cells, and it can be blocked by estrogen receptor antagonists (Chan et al., 2002). Rg1 can also improve nonspecific immunity in humans and promote the hematopoietic and immune function recovery of patients with bone marrow injury; thus, this molecule can be used to treat various immune and hematopoietic system diseases (Lee et al., 2004; Xu et al., 2012). Simultaneously, five clinical trials on the use of drugs containing Rg1 to treat vascular dementia, cognitive changes, Sjögren's syndrome, rheumatic diseases, and stroke, as well as a safety evaluation, have been registered on clinicaltrials.gov; three of these trials have completed recruitment, and the related results have been published; two have not yet completed subject recruitment (Sotaniemi et al., 1995; Ellis and Reddy, 2002; Scholey et al., 2010; Ossoukhova et al., 2015; Shin et al., 2016; Tian et al., 2016).

In recent years, the characteristics, functions, and therapeutic effects of MSCs and the pharmacological effects of Rg1 have been extensively studied (Zhan et al., 2014; Shyh-Chang and Ng, 2017; Jin et al., 2019). The effect and mechanism of Rg1 on the biological characteristics and functions of MSCs is becoming increasingly clear. Multiple studies have found that Rg1 regulates the proliferation, differentiation, aging, and apoptosis of MSCs and thus affects tissue repair in the body.

## Optimization of the Effective Concentration of Rg1

Appropriate concentrations of Rg1 can effectively regulate the expression of functional proteins and the secretion of active cytokines in MSCs, and overdosages can cause toxicity to cells and tissues (Liu et al., 2005; Mohanan et al., 2018). Traditionally, the active ingredients in ginseng are believed to be good nutritional supplements for pregnant women and beneficial for fetal development (Tiran, 2003; Ong et al., 2005). Recent studies have found that some concentration of Rg1 may have embryotoxic effects (Liu et al., 2006; Mohammed et al., 2016). In studies using the whole embryo culture technique, culturing with Rg1 (62.4 mM for mice and 37.4 mM for rats) for 48 h reduced the total embryo morphological score, which is based on the crown-rump length, head length, flexion scores, forelimb bud scores, and hindlimb bud scores. Furthermore, the development of the heart; neural tube; cerebral vesicles; otic, optic, and olfactory organs; branchial arch; maxilla; mandible; yolk sac vasculature; and allantois was also affected by increased concentrations of Rg1 (Liu et al., 2006). In contrast, a low concentration of Rg1 (62.5–10000 nM) may have a slight effect on chick cardiomyocytes and mouse D3 stem cells (Mohammed et al., 2016). Therefore, pregnant women should be cautious when using ginseng or ginsenoside Rg1 during the first three months of pregnancy. This molecule should be administered at low concentrations. Studies have also shown that 1,000 μg/L Rg1 inhibits the proliferation of rat bone marrow MSCs (BMSCs) (Fu and Zheng, 2013), and Rg1 concentrations exceeding 100 mol/L are cytotoxic to human periodontal ligament stem cells (hPDLSCs) (Yin et al., 2015). Therefore, systematic and comprehensive studies need to be performed to clarify the toxic effects of different concentrations of Rg1 on human MSCs. In addition, we evaluated and classified the effective, ineffective, and inhibitory properties of Rg1 on the proliferation of MSCs *in vitro* and summarized and analyzed the effects of different Rg1 concentrations, hoping to provide a reference for subsequent studies (**Supporting Information 1**).

## Rg1 Regulates the Proliferation and Differentiation of MSCs

**Figure 2:**
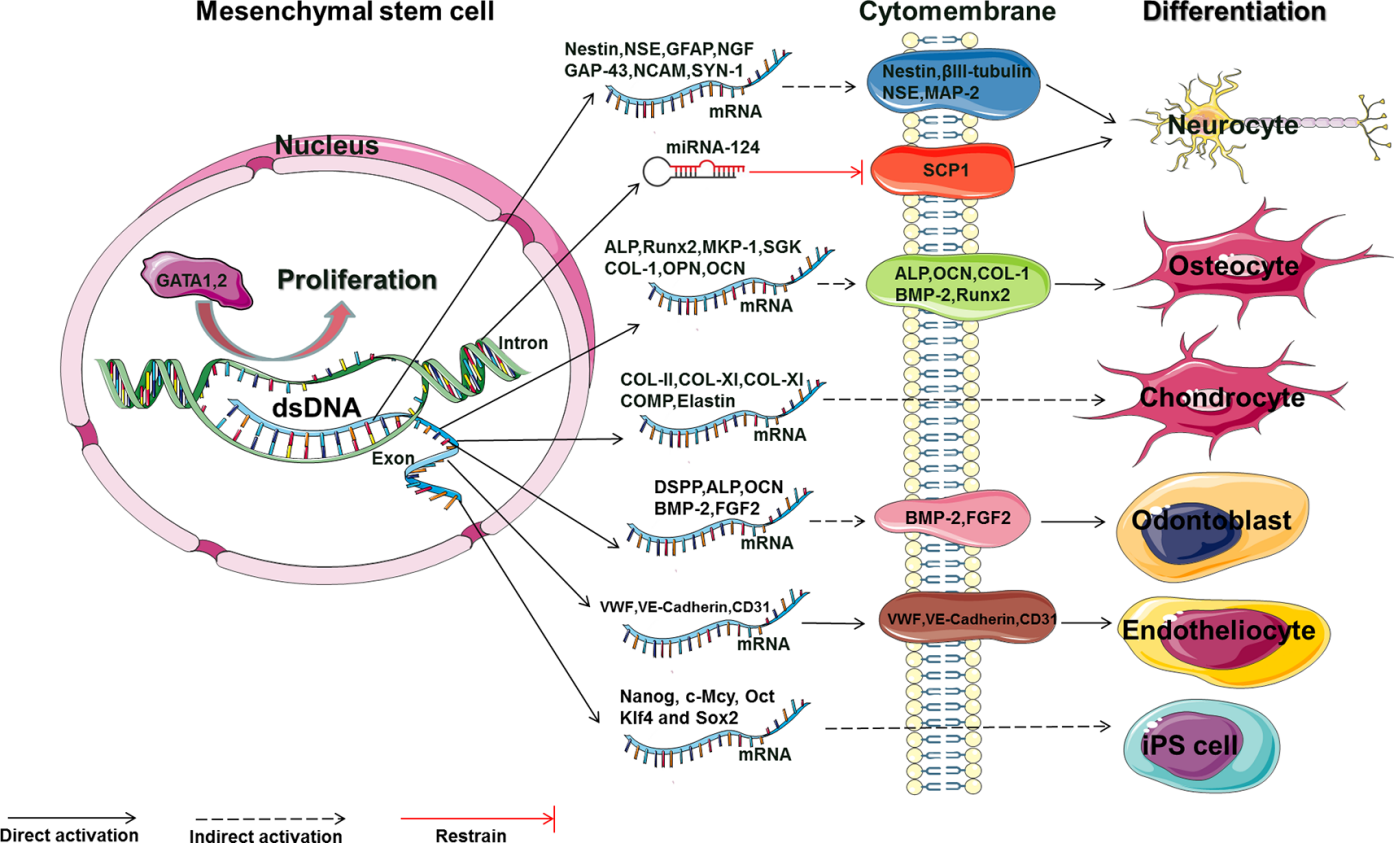
The main molecular mechanism by which Rg1 affects the proliferation and differentiation of MSCs (GATA, GATA-binding factor; NSE, neuron-specific enolase; GFAP, glial fibrillary acidic protein; NGF, nerve growth factor; GAP-43, growth-associated protein 43; NCAM, neural cell adhesion molecule; SYN, synaptophysin; MAP-2, microtubule-associated protein 2; SCP-1, small C-terminal domain phosphatase 1; ALP, alkaline phosphatase; OCN, osteocalcin; COL, collagen; BMP-2, bone morphogenetic protein 2; RUNX2, runt-related transcription factor 2; COMP, cartilage oligomeric matrix protein; DSPP, dentin sialophosphoprotein; BMP-2, bone morphogenetic protein 2; FGF2, fibroblast growth factor 2; vWF, von Willebrand factor; VE-cadherin, vascular endothelial cadherin; Nanog, c-Mcy, Oct, Klf4 and Sox2: embryonic stem cell-related transcription factors).

Conventionally, MSCs are separated and purified by adhering to plastic and undergoing serial passaging *in vitro* to expand the primary cells (Bruder et al., 1997; Le Blanc and Ringden, 2007). For the MSC drug preparation process, the MSC proliferation rate must be increased and sufficient amounts of purified MSCs must be obtained (Ikada, 2006; Ikebe and Suzuki, 2014). Furthermore, enhancing the differentiation of MSCs into neurocyte cells, endothelial cells, or bone cells will contribute to the treatment of clinical diseases, such as neuronal disease, cardiovascular disease, and bone injury (Deans and Moseley, 2000). At present, studies have shown that Rg1 can positively regulate the proliferation of MSCs from various adult tissues (**Table 1**) and induce the directional differentiation of MSCs in different environments (**Table 2**, **Figure 2**).

**Table 1:** Ginsenoside Rg1 promotes MSC proliferation *in vitro*.

Study	MSC source	Proliferation	Notes
Dong et al. (2017)	Mouse adipose tissue	MTT↑	Cells during isobutylmethylxanthine neural induction
Gu et al. (2016)	Rat bone marrow	EdU+ cells, CCK8↑	
Hu et al. (2016)	Rat bone marrow	CCK8↑	Cells inhibited by H_2_O_2_
Liu et al. (2016)	Human umbilical cord blood	Colony-forming unit of fibroblasts (CFU-F), CCK8↑	Cells inhibited by tert-butyl hydroperoxide
Xu et al. (2016)	Human adipose tissue	CCK8↑	Rg1 combined with platelet-rich fibrin
Xu et al. (2014)	Human adipose tissue	CCK8↑	Cells during the neurogenic differentiation process
Xu et al. (2015)	Human adipose tissue	CCK8↑	Cells during the chondrogenic induction process
Yin et al. (2015)	Human periodontal ligament	MTT↑	Cells during dexamethasone osteogenic induction
Wang et al. (2014)	Human dental pulp	G0/G1↓; [3H]-Thymidine incorporation assay, S phase↑,	
Wang et al. (2012)	Human dental pulp	G0/G1↓; colony-forming unit, MTT, S phase↑	
Wang et al. (2007)	Rat bone marrow	CFU-F, [3H]-thymidine incorporation assay, GATA1 and GATA2, binding activities of GATA and DNA, MTT↑	
Guo et al. (2017)	Human bone marrow	MTT↑	Rg1-loaded alginate-chitosan microspheres
Fu and Zheng (2013)	Rat bone marrow	MTT↑	Cells during hypoxia/serum deprivation

**Table 2:** Ginsenoside Rg1 promotes the directional differentiation of MSCs *in vitro*.

Study	MSC source	Differentiation effects	Related mechanism
Dong et al. (2017)	Mouse adipose tissue	Neural differentiation induced by isobutylmethylxanthine↑	Small C-terminal domain phosphatase 1↓; miRNA-124, Nestin, β-tubulin III↑
Xu et al. (2014)	Human adipose tissue	Neural differentiation in neural inductive conditioned medium↑	NSE^a^, microtubule-associated protein-2, growth-associated protein-43, neural cell adhesion molecule, synapsin-1↑
Wu et al. (2011)	Rat bone marrow	Neural differentiation inhibited by the brain homogenate of a rat dementia model↑	NSE^a^ positive cells↑
Zuo et al. (2007)	Rat bone marrow	Neural differentiation↑	NSE^a^, nerve growth factor↑
Guo et al. (2017)	Human bone marrow	Rg1-loaded alginate-chitosan microspheres promote neural differentiation	Nestin, NSE^a^, glial fibrillary acidic protein↑
Gu et al. (2016)	Rat bone marrow	Osteogenic differentiation↑	ALP^b^, Alizarin red staining, RUNX2, BMP-2, collagen I, osteocalcin↑; regulate the GR-dependent BMP/Smad pathway
Yin et al. (2015)	Human periodontal ligament	Osteogenic differentiation in dexamethasone induction↑	ALP^b^ activity, RUNX2^c^, collagen I, osteopontin, osteocalcin↑
Wang et al. (2014)	Human dental pulp	Odontogenic/osteogenic differentiation↑	Dentin sialoprotein, DSPP^d^, ALP^b^, osteocalcin, bone morphogenetic protein-2, fibroblast growth factor 2↑
Huang et al. (2016)	Mouse bone marrow	Osteogenic differentiation inhibited by malondialdehyde↑	calcium nodule, ALP^b^, Runx2^c^↑
Xu et al. (2015)	Human adipose tissue	Cartilage differentiation in osteogenic induction medium↑	Collagen II, collagen XI, acid phosphatase, cartilage oligomeric matrix protein, ELASTIN↑
Wang et al. (2012)	Human dental pulp	Odontoblast-like cells↑	ALP^b^; DSPP^d^, DMP1^e^↑
Wang and Yaping (2012)	Human dental pulp	Rg1 combined with rhBMP-2 promotes odontogenic differentiation	ALP^b^ activity, DSPP^d^, DMPl^e^↑
He et al. (2011)	Human bone marrow	Endothelial differentiation in an indirect coculture with human umbilical vein endothelial cells↑	Von Willebrand factor, VE-cadherin, CD31↑
Li et al. (2011)	Rat bone marrow	Induced pluripotent stem cell differentiation↑	Nanog, c-Myc, Oct, Klf4, Sox2↑

a NSE : Neuron-specific enolase.

b ALP : Alkaline phosphatase.

c RUNX2 : Runt-related transcription factor 2.

d DSPP : Dentin sialophosphoprotein.

e DMP1 : Dentin matrix protein 1.

Initially, bone marrow was used as the main source to isolate MSCs, and methods for harvesting BMSCs are highly invasive. The limited number and maximal life span of primary MSCs harvested from bone marrow is a major challenge in the clinical use of these cells (Jiang et al., 2002; Kern et al., 2006). In previous experiments, Rg1 could promote the proliferation of rat BMSCs by upregulating the expression of GATA1 and GATA2 and enhancing their binding to DNA (Wang et al., 2007). Reports have also shown that 10 μmol/L Rg1 can promote the neuron-specific enolase (NSE) expression of rat BMSCs in low-glucose Dulbecco's modified Eagle's medium (DMEM) with or without brain homogenates from rats with dementia; furthermore, this treatment prevented the brain homogenate (dementia rats) induced inhibition of neuronal differentiation and enhanced the differentiation of BMSCs into specific subpopulations of neuron-like cells that express nerve growth factor (NGF) mRNA (Zuo et al., 2007; Wu et al., 2011). Moreover, Rg1-loaded alginate-chitosan microspheres were also shown to promote the proliferation and neuron-like differentiation of human BMSCs, increasing the expression of Nestin, NSE, and GFAP (Guo et al., 2017). This finding suggests that Rg1 can be used in the drug delivery/release studies and tissue engineering. However, no studies have explored the detailed mechanism by which Rg1 induces MSCs to differentiate into neuron-like cells with specific functions. For example, studies investigating the regulatory effects of Rg1 on BMSC differentiation into dopaminergic neurons or cholinergic neurons are needed. Therefore, clarification of the regulatory methods and related mechanisms of Rg1 may also be a “breakthrough point” for the treatment of neuronal disease such as Parkinson's disease and Alzheimer's disease and related neurological disorders *in vivo*.

Rg1 can also promote the differentiation of human BMSCs into endothelial cells. Experiments involving BMSCs and human umbilical vein endothelial cells cocultured in a transwell system showed that 20 to 80 μg/ml Rg1 gradually increased the mRNA and protein levels of mature endothelial cell-specific markers in BMSCs, such as CD31, VWF, and VE-cadherin, in a dose-dependent manner. At the same time, electron microscopy showed that the characteristic endothelial Weibel-Palade bodies were observed in BMSCs (He et al., 2011). Animal experiments *in vivo* have also confirmed that Rg1 can stimulate the myocardial tissue secretion of granulocyte colony-stimulating factor (G-CSF), which promotes the homing of rabbit BMSCs to myocardial tissue and differentiation into vascular endothelial cells. These changes reduced the myocardial infarct size and improved cardiac function (Wang et al., 2005). Notably, restenosis is the most common complication of stent surgery for cardiovascular diseases, and its pathological mechanism involves the delayed formation of vascular endothelial cells and the proliferation of vascular smooth muscle cells (Nobuyoshi et al., 1991; Ferns and Avades, 2000; Tonino et al., 2009). The abovementioned study showed that Rg1 can promote the differentiation of MSCs into vascular endothelial cells. Thus, loading Rg1 or MSCs onto vascular balloons (Schwartz et al., 1990) or stents (Hoffmann et al., 1996; Nakatani et al., 2003) may be an effective method to promote endothelial cell regeneration. Besides, whether Rg1 can combine with MSCs to inhibit the proliferation of vascular smooth muscle cells remains unknown. Therefore, demonstrating that Rg1 combined with MSCs can enhance endothelial cell proliferation while inhibiting smooth muscle cell proliferation will support this hypothesis and provide a strategy for restenosis therapy.

Rg1 can promote the osteogenic differentiation of rat BMSCs and has a protective effect on the malondialdehyde-inhibited osteogenic differentiation of mouse BMSCs. The molecular mechanism involved increased formation of calcium nodules and alkaline phosphatase (ALP) activity and upregulation of the mRNA and protein levels of ALP and Runt-related transcription factor 2 (Runx2) (Gu et al., 2016; Huang et al., 2016). In addition, Rg1 can mobilize glucocorticoid receptors to form homodimers and transfer to the rat BMSC nucleus, promoting the increased transcription of glucocorticoid receptor-sensitive genes, such as mitogen-activated protein kinase phosphatase-1 (MKP-1) and glucocorticoid-regulated kinase (SGK). Then, bone morphogenetic protein-2 (BMP-2) is activated to increase the expression of p-Smad1/5/8 (glucocorticoid receptor-dependent BMP-2/Smad signaling pathway); upregulates the protein expression of osteogenic proteins such as ALP, CON, COL1, BMP-2, and Runx2 in BMSCs; enhances bone calcification; and finally, promotes bone healing in rats (Gu et al., 2016). The positive effect of Rg1 on the osteogenic differentiation of BMSCs has been established. However, periosteal tissues and periosteal stem cells are the main mobilization components involved in bone repair after a fracture (Tonna, 1960; Debnath et al., 2018). How Rg1 promotes the differentiation of BMSCs into bone cells in the bone “stem cell niche” to promote fracture healing still needs to be elucidated. Furthermore, whether the intraperitoneally injected Rg1 described in the above study mainly exerts osteogenic and bone-healing effects by directly acting on periosteal stem cells in rats requires further confirmation.

In α-MEM culture, addition of Rg1 (6.25 μmol/L) to cultured rat BMSCs for 30 days increased the mRNA levels of Nanog, c-Mcy, Oct, Klf4, and Sox2. Some scholars believe that Nanog-positive cells and embryonic stem cells are difficult to distinguish *via* their gene expression profiles. Therefore, based on gene expression analyses, they concluded that Rg1 may promote the conversion of BMSCs to induced pluripotent stem cells (iPSs) (Takahashi et al., 2007; Li et al., 2011). However, it is difficult to explain the iPS-promoting effect of Rg1 based only on gene expression. At the same time, one study also showed that 1 μg/ml Rg1 can effectively increase the efficiency of mouse embryonic fibroblast conversion to iPSs (Hu et al., 2014). The success rate of Rg1-induced iPSs and the tumorigenicity and safety of the induced iPSs in the above studies require further analyses.

Compared with BMSCs, adipose-derived MSCs (ADSCs) are easy to obtain and result in less trauma during tissue extraction, indicating that they are ideal seed cells for tissue engineering (Puissant et al., 2005; Kern et al., 2006; Bourin et al., 2013). Rg1 and platelet fibrin (PRF) can enhance the proliferation, differentiation, and soft tissue regeneration of human breast adipose ADSCs in collagen type I sponge scaffolds *in vitro* and *in vivo*. Three weeks after the subcutaneous transplantation of Rg1 in combination with PRF-treated human breast ADSC-loaded type I sponge scaffolds, the “adipose tissue block” wet weight, number of adipocytes, intracellular lipid levels, microvascular density, and gene and protein levels of VEGF, HIF-1α, and PPARγ were significantly improved, and a broad new organizational network was also formed. These results suggested that Rg1 and PRF combined with 3D culture can enhance the effects of MSCs in soft tissue regeneration in tissue engineering studies (Xu et al., 2016). However, the occurrence of related risk factors, such as tumors and cancer, must be considered in the formation of various new tissues. For instance, RUNX2 is a transcription factor belonging to the RUNX family and plays a key role in osteoblast differentiation (Komori, 2018). This molecule is also considered a marker of MSCs found in tumors and is involved in various signaling pathways of cancer growth processes (Ozaki et al., 2018). New research has found that lyophilized PRF can mediate Runx2-enhanced craniofacial bone regeneration (Li et al., 2014). Furthermore, Rg1 can stimulate human umbilical vein endothelial cells to undergo angiogenesis *via* the miR-23a/RUNX2/VEGF-A pathway (Wu et al., 2017). Therefore, key factors, such as the presence of bone tissue components in the “adipose tissue block” induced by PRF and Rg1 and the correlation between Runx2 expression in transplanted adipose stem cells and tumor formation, must be clarified before clinical trials.

Rg1 can increase the proliferation and neurodifferentiation of human ADSCs. In human ADSCs grown in nerve-inducing medium, Rg1 at concentrations of 10, 50, and 100 μg/ml can gradually increase proliferation; enhance the gene transcription levels of growth-associated protein 43 (GAP-43), neural cell adhesion molecule (NCAM), and synapse protein 1 (SYN-1); and increase the protein expression levels of NSE and microtubule-associated protein-2 (MAP-2) (Xu et al., 2014). In addition, Rg1 can increase the expression of miRNA-124, degrade the protein expression of the neuronal differentiation inhibitor small C-terminal domain phosphatase 1, and increase the protein levels of nestin and βIII-tubulin, thereby promoting the differentiation of ADSCs into nerve-like cells after induction by isobutylmethylxanthine (IBMX) (Dong et al., 2017). In cartilage-inducing medium, Rg1 can enhance the proliferative capacity of third-generation human breast ADSCs in the early stage (6 days after seeding) and promote the phenotypic differentiation of chondrocytes in the late phase (2 weeks after seeding) by, for example, enhancing the mRNA levels of collagen type II (CO-II), collagen type XI (CO-XI), acid phosphatase (CO-XI), cartilage oligomeric matrix protein (COMP), and ELASTIN (elastin). Researchers have suggested that third-generation human ADSCs can be used for *in vitro* cartilage regeneration and that the addition of Rg1 at different time points can effectively regulate the proliferation and cartilage differentiation of human breast ADSCs (Xu et al., 2015).

Rg1 can promote the proliferation of dental pulp and PDLSCs, the differentiation of human dental pulp stem cells (DPSCs) into odontoblasts, and the differentiation of human PDLSCs into osteoblasts, indicating its promising application prospects in the field of dental and oral and maxillofacial regenerative medicine. Rg1 can promote the proliferation and differentiation of human DPSCs *via* 2059 differentially expressed genes; for example, Rg1 increases the gene expression of DSPP, DMP1, ALP, OCN, BMP-2, and FGF2 and the protein expression of BMP-2 and FGF2 in DPSCs (Wang et al., 2012, 2014). At the same time, the combination of Rg1 and rhBMP-2 can enhance the odontogenetic differentiation of human DPSCs (Wang and Yaping, 2012). Therefore, the application of Rg1 as a pulp capping agent in clinical pulp capping surgery may be a new breakthrough for the promotion of DPSC proliferation and differentiation, which will be beneficial for regenerating dental pulp and promoting the subsequent formation of reparative dentin. A 10 µmol/L Rg1 solution can increase the proliferation of PDLSCs and enhance the ALP, RUNX2, collagen I, osteopontin (OPN), and osteocalcin (OCN) expression in hPDLSCs, ultimately promoting the osteogenic differentiation of human PDLSCs. However, concentrations of Rg1 exceeding 100 μmol/L inhibited cell proliferation (Yin et al., 2015). Interestingly, dental pulp, periodontal ligaments, dentin, cementum, and intrinsic alveolar bone all originate from ectodermal mesenchymal tissue (exo-mesenchymal), which is developed from ectodermal neural crest cells. Nerve-supporting cells or neurogliocytes (Schwann cells and Schwann cell precursors, etc.) transfer to the dental pulp tissues during early development to form dental pulp MSCs (Kaukua et al., 2014). This phenomenon indicates that the MSCs stored in the connective tissue of the oral cavity, especially in dents, may be different from traditional mesenchymal-derived MSCs and may show enhanced neurological differentiation.

## Rg1 Relieves the Aging of MSCS

With the increase in MSC passage number *in vitro* and the aging of human organs, telomere length shortens after each division cycle, which leads to gradual senescence (Fehrer and Lepperdinger, 2005; Alt et al., 2012). The mean telomere length of BMSCs decreased from 9.19 kbp to 8.7 kbp from passages 1 to 9 (Bonab et al., 2006). Mesenchymal and hematopoietic stem cells can form a unique bone marrow niche, the members of which are closely related and influence each other (Wilson and Trumpp, 2006; Méndez-Ferrer et al., 2010). Rg1 can enhance the antiaging effects of hematopoietic stem cells and the hematopoietic microenvironment (partly by regulating the secretion of MSCs), prevent cognitive impairment and hippocampus senescence, antagonize spleen and thymus damage induced by D-galactose (D-gal), and ameliorate induced aging animal models by alleviating oxidative stress injury and downregulating the expression of senescence-associated proteins (Zhu et al., 2014; Tang et al., 2015; Sun et al., 2018). Similarly, Rg1 has antiaging effects on MSCs and cooperates with other supporting cells to protect tissues and organs. After rats were treated with D-gal and Rg1, BMSCs were extracted from the bone marrow for culture. In three-passage cultures, the MSCs isolated from Rg1-treated rats showed enhanced antioxidant and anti-inflammatory properties and a strong ability to resist hematopoietic microenvironment senescence, exhibiting features such as a reduced percentage of SA-β-gal+ cells, reduced reactive oxygen species (ROS) levels, reduced malondialdehyde (MDA) activity, and reduced expression of inflammatory markers (IL-6, IL-2, TNF-α) and senescence-associated proteins (p16, p21, p53) as well as an increased S phase cell percentage, increased superoxide dismutase (SOD) activity, and increased stem cell factor (SCF) expression. At the same time, Rg1 also showed increased antioxidant and partial anti-inflammatory properties in normal rats (Hu et al., 2015). These results indicate that Rg1 can regulate MSCs to affect the microenvironment and thus enhance hematopoiesis. This finding suggests a new strategy for addressing the insufficient hematopoietic stem cell quantity, the low homing rate of transplanted hematopoietic stem cells, and the delayed implantation of hematopoietic stem cells in the bone marrow during hematopoietic stem cell transplantation.

Furthermore, the fact that different culture environmental conditions can also affect telomeres and significantly increase or decrease the culture life span *in vitro* (von Zglinicki et al., 1995) still needs to be addressed. Thus, the culture and treatment of MSCs with Rg1 may minimize the effect of serum-free medium, the culture temperature, and the oxygen content on aging *in vitro*, which need to be investigated before MSCs can be used therapeutically.

## Rg1 Inhibits MSC Apoptosis

To maintain a stable environment in the body, genes control the autonomous and orderly death of cells (Wyllie et al., 1980; Schwartz and Osborne, 1993). A study showed that more than 99% of MSCs injected into the left ventricle of CB17 SCID/beige adult mice died within 4 days of injection, which reflects the harsh, proapoptotic microenvironment of the infarcted heart; this environment may not be conducive to MSC survival (Geng, 2003). Hence, protection of MSCs from apoptosis may improve their success rate in treating tissue ischemia and hypoxia-related diseases (Zhu et al., 2006).

**Figure 3:**
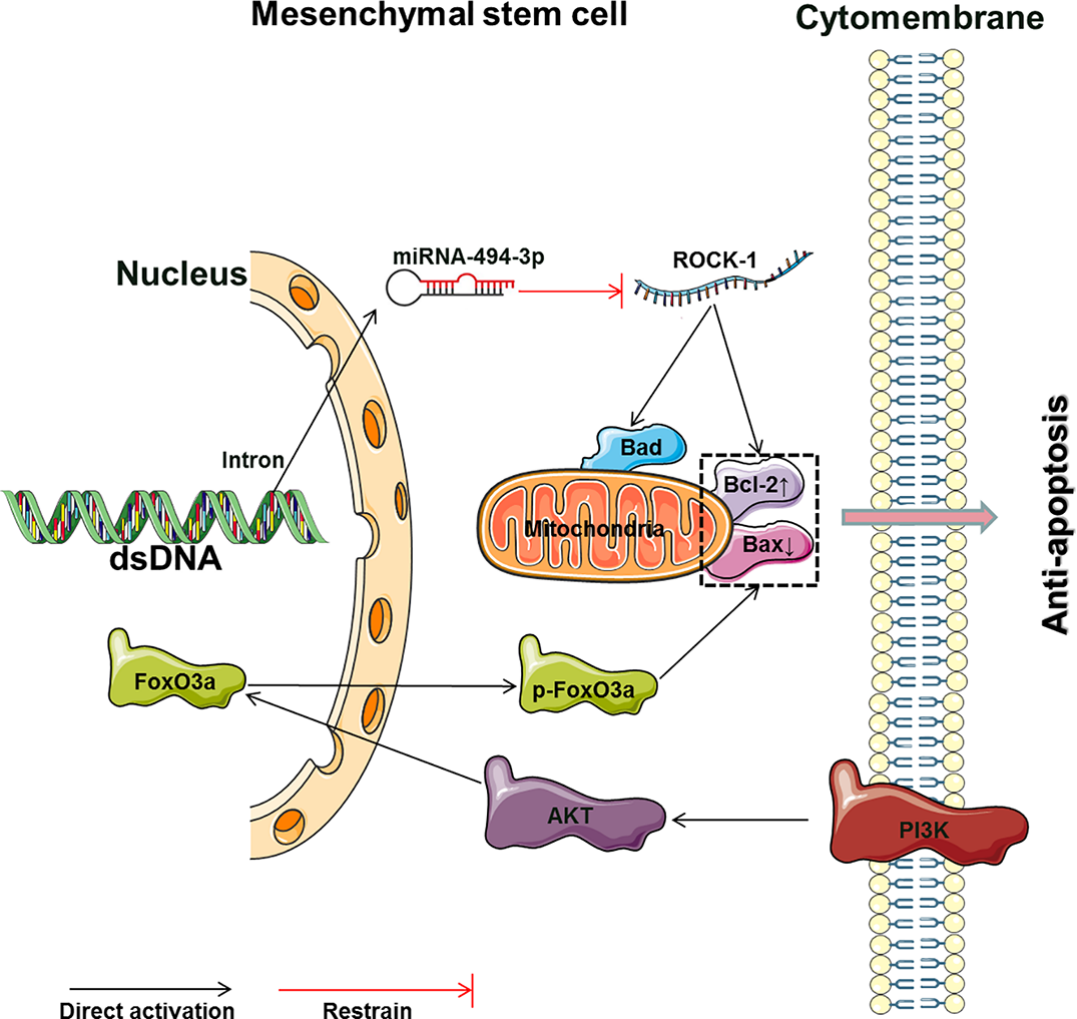
The main molecular mechanism by which Rg1 affects the apoptosis of MSCs (Bad, Bcl2-associated agonist of cell death; Bcl-2, B cell lymphoma 2; Bax, B cell lymphoma/leukemia-2-associated X protein; ROCK-1, Rho-associated coiled-coil containing protein kinase 1; PI3K, phosphoinositide 3-kinases; AKT, protein kinase B; FoxO3, forkhead box O3; p-FoxO3, phosphorylated forkhead box O3).

Rg1 has extensive antiapoptotic effects in different apoptotic models (**Table 3**, **Figure 3**). Rg1 can reduce apoptosis and increase the water content in the brain tissues of ischemia-reperfusion rats. Transplanted human BMSCs in rats can differentiate into neurons and neurogliocytes to improve cerebral ischemia. More importantly, Rg1 combined with human BMSCs showed better antiapoptotic effects and resulted in better brain recovery than single Rg1 or BMSC treatments (Bao et al., 2015). Rg1 can counteract the apoptosis of BMSCs induced by various stimuli, and the expression levels and ratio of Bcl-2/Bax are key factors in the Rg1-induced resistance of BMSCs to apoptosis. Rg1 can reduce Bax expression and enhance Bcl-2 expression to protect against hypoxia-reoxygenation-induced apoptosis of human BMSCs (Guo et al., 2017), MDA-induced apoptosis of mouse BMSCs (Li et al., 2013), and hypoxia and serum deprivation-induced apoptosis of rat BMSCs (Fu and Zheng, 2013). Rg1 can also activate the phosphoinositide 3-kinase/protein kinase B (PI3K/Akt) signaling pathway to protect against the hydrogen peroxide (H_2_O_2_)-induced apoptosis of rat BMSCs. The mechanism involves downregulating Bax, cleaving caspase-3, and upregulating Bcl-2 and phosphorylated Akt at the protein level (Hu et al., 2016). Furthermore, in an *in vivo* experiment involving the *in situ* injection of Rg1-treated rat BMSCs to treat hindlimb arterial embolization in rats, Rg1 improved the survival of transplanted BMSCs and protected BMSCs from apoptosis induced by ischemia. The Rg1 mechanisms in the above study involve the activation and upregulation of miR-494-3p to inhibit ROCK-1 gene expression, upregulation of the antiapoptotic gene Bcl-2, downregulation of the proapoptotic genes Bax and Bad, inhibition of the Bax and Bcl-2 protein interaction to prohibit heterodimer formation, and inhibition of the release of cytochrome C from mitochondria and caspase-3 activation (Zheng et al., 2018).

**Table 3:** Ginsenoside Rg1 inhibits MSC apoptosis *in vitro*.

Study	MSC source	Reagent for cell apoptosis	Effects	Related mechanism
Gu et al. (2016)	Rat bone marrow	Dexamethasone?	Inhibit apoptosis; Annexin V/propidium iodide↓	
Hu et al. (2016)	Rat bone marrow	H_2_O_2_	inhibit apoptosis; TUNEL staining, Annexin V/propidium iodide↓	Cleaved caspase-3/total caspase-3↓; Bcl-2/Bax, p-Akt/Akt↑
Liu et al. (2016)	Human umbilical cord blood	Tert-Butyl hydroperoxide	Inhibit apoptosis; Annexin V-FITC/7-AAD, TUNEL-positive cells, condensation and fragmentation of nuclei↓	Activate the Akt-FoxO3a-Bim pathway; LDH, MDA, cleaved caspase-3, Bim, FoxO3a (nucleus)↓; Bcl-2/Bax, SOD, pAKT/AKT, pFoxO3a/FoxO3a, FoxO3a (cytoplasm)↑
Guo et al. (2017)	Human bone marrow	Hypoxia-reoxygenation	Inhibit apoptosis	Bcl-2↓; Bax↑
Li et al. (2013)	Mouse bone marrow	Malondialdehyde	Rg1-loaded alginate-chitosan microspheres inhibit apoptosis; TUNEL-positive cells↓	Bax, caspase-3↓; Bcl-2↑
Fu and Zheng (2013)	Rat bone marrow	Hypoxia/serum deprivation	Inhibit apoptosis; Annexin V-FITC/propidium iodide↓	Stable mitochondrial membrane structure and function; caspase-3 activity, Bad, Bax↓;Bcl-2↑

Tert-butyl hydroperoxide (t-BHP) was shown to reduce the Bcl-2/Bax ratio and stimulate ROS formation to enhance apoptosis (Kowaltowski and Fiskum, 2005; Lee et al., 2012). Rg1 can enhance the survival rate and antiapoptotic effects of human umbilical cord blood-derived stromal cells (hUCBDSCs) induced by t-BHP. The mechanism involves activation of the PI3K-Akt pathway and induction of FoxO3a phosphorylation, resulting in the translocation of FoxO3a from the nucleus to the cytoplasm; these changes reduce Bim expression and increase the Bcl-2/Bax ratio to inhibit apoptosis (Liu et al., 2016). Interestingly, MSCs can reside in a state of reversible growth arrest, or quiescence, for prolonged periods of time, and cells in this state are called “idle” stem cells (van Velthoven and Rando, 2019). Do nonapoptotic MSCs protected by Rg1 remain in an “idle” state? Clarifying this question may explain the mechanism underlying the functional maintenance of quiescent “idle” MSCs and activation of MSCs to modify tissue homeostasis in a specific environment, such as hypoxia and ischemia.

## Conclusions and Remarks

We comprehensively visualized the main molecular mechanism by which Rg1 affects the proliferation, differentiation, and apoptosis of MSCs described in the above papers (**Figures 2** and **3**). This result may provide a useful reference and ideas for further experimental research on Rg1. In summary, Rg1 shows promising application prospects with regard to MSCs and its microenvironmental regulatory effects *in vitro* and in several animal disease models *in vivo* (**Table 4**). During the expansion of pretreatment MSCs *in vitro*, various uncontrollable factors, such as differences in culture systems, culture environment, and reagents, may be encountered, which may affect the MSC quality before clinical infusion or use in tissue engineering. The application of Rg1 may provide an effective solution for these issues. Furthermore, although current studies have shown that Rg1 can effectively regulate the proliferation and differentiation of MSCs while controlling senescence and apoptosis *in vivo*, how to minimize the toxicity of Rg1, how Rg1 functions in a specific “stem cell niche,” how to utilize Rg1 to precisely regulate MSC-directed differentiation in the microenvironment, and how Rg1 affects the balance between “idle” and activated MSC populations to maintain homeostasis or repair the human body still require additional, in-depth studies.

**Table 4:** Animal experimental studies of ginsenoside Rg1 regulating MSCs *in vivo*.

Study	Animal model	Rg1 dosage	Administration methods	Study object	Therapeutic effect	Related mechanism
Xu et al. (2016)	Untreated nude mice	10 μg/ml	Collagen scaffold-loaded adipose MSCs plus Rg1 or/and PRF mouse subcutaneous transplantation	Scaffold transplant	Wet weight of the transplant, adipogenesis, microvessel density↑	PPARγ, HIF-1α, VEGF↑
Gu et al. (2016)	Rat tibial fracture model	20 mg/kg per day	Rg1 intraperitoneal injection	Fractured tibia	Improved H&E staining, Safranin-O/Light Green Red staining, bone mineral density, bone volume, trabecular number, trabecular separation	
Hu et al. (2015)	D-Galactose-induced aged rat model	20 mg/kg per day	Rg1 intraperitoneal injection of rats and extracted BMSCs for analysis	BMSCs from aged rats		SA-β-gal+ cell%, ROS, MDA, IL-2, IL-6, TNF-α, p16^INK4a^, p21^Cip1/Waf1^, p53 pro↓; cell proliferation, S phase%, SOD activity, stem cell factor↑
Hu et al. (2015)	Normal rats	20 mg/kg per day	Rg1 intraperitoneal injection of rats and extracted BMSCs for analysis	BMSCs from aged rats		SA-β-gal+ cell%, G1 phase %, ROS, MDA, IL-6,p16↓; cell proliferation, TNF-α, S phase %, SOD activity↑
Bao et al. (2015)	MCAO and reperfusion models (intraluminal vascular occlusion method)	20 mg/kg per day	Rat BMSC venous transplantation followed by Rg1 intraperitoneal injection	Rat ipsilateral and contralateral hemispheres	Brain edema, infarct volume↓; neurological outcome↑	TUNEL staining, Bax pro↓; neuron-specific enolase, glial fibrillary acidic pro, Bcl-2 pro, Bcl-2/Bax↑
Zheng et al. (2018)	Hind limb ischemia model	100 μg/ml (cultured with BMSCs)	BMSCs treated with Rg1 orthotopic transplantation	Gastrocnemius and tibialis anterior muscle	Improved the survival of transplanted BMSCs and enhanced the therapeutic effects	ROCK-1, MLC-2, Bad, Bax↓; Bcl-2, miR-494-3p↑
Wang et al. (2005)	Rabbit myocardial infarction model	10 mg/(kg·d)	Rabbit BMSC iliac transplantation followed by Rg1 treatment	Rabbit heart	Area of myocardial infarction↓; cardiac function; regeneration of myocardial endothelial cells↑	Granulocyte colony-stimulating factor in cardiac muscle↑

## Author Contributions

Conceptualization: FH. Methodology: FH and TL. Software: FH. Validation: FH. Formal analysis: FH and CY. Investigation: FH and HJ. Resources: FH. Data curation: FH. Writing—original draft preparation: FH. Writing—review and editing: FH and CY. Visualization: FH. Supervision: FH. Project administration: FH and TL. Funding acquisition: FH.

## Funding

This work was supported by the Master's Startup Fund of Zunyi Medical College (Yuan Zi [2017] No. 29) and the Traditional Chinese Medicine and Ethnic Medicine Science and Technology Research Project of Guizhou Province (QZYY-2018-113).

## Conflict of Interest

The authors declare that the research was conducted in the absence of any commercial or financial relationships that could be construed as a potential conflict of interest.
