# Supplementary material for: Ginsenoside Rg1 as an Effective Regulator of Mesenchymal Stem Cells
Source: Front Pharmacol. 2020 Jan 23;10:1565. doi: 10.3389/fphar.2019.01565 (PMC6989539; doi:10.3389/fphar.2019.01565)
Supplement: Supplementary file 1 [file Table_1.docx]

**Supporting Information 1.** The effective, ineffective and inhibitory effects of different concentrations of Rg1 on the proliferation of MSCs in vitro.

**Supporting information 1**

| N | Mesenchymal stem cells | Time | Valid (μM) | Invalid (μM) | Toxicity (μM) | Notes | References |
| --- | --- | --- | --- | --- | --- | --- | --- |
| 1 |  |  |  |  |  |  |  |
| 2 | Mouse adipose stem cells | 24 h | 62.421 124.842 | 12.4842 |  | Cells cultured with isobutylmethylxanthine | Dong et al. (2017) |
| 3 | Rat bone marrow mesenchymal stem cells | 24 h |  | 0.1248 1.24842 12.4842 |  | Cells treated with dexamethasone | Gu et al. (2016) |
| 4 | Rat bone marrow mesenchymal stem cells | 48 h | 1.24842 | 0.1248 12.4842 |  | Cells treated with dexamethasone | Gu et al. (2016) |
| 5 | Rat bone marrow mesenchymal stem cells | 72 h | 0.1248 1.24842 12.4842 |  |  | Cells treated with dexamethasone | Gu et al. (2016) |
| 6 | Rat bone marrow mesenchymal stem cells | 96 h | 0.1248 1.24842 12.4842 |  |  | Cells treated with dexamethasone | Gu et al. (2016) |
| 7 | Rat bone marrow mesenchymal stem cells | 24 h | 10 100 | 0.1 1 |  | Cells treated with H2O2 | Hu et al. (2016) |
| 8 | Human umbilical cord blood stem cells | 24 h | 1 10 50 | 0.01 0.1 |  | Cells treated with tert butyl hydroperoxide | Liu et al. (2016) |
| 9 | Human adipose stem cells | 24 h |  | 12.4842 |  | Cells combined with platelet rich fibrin | Xu et al. (2016) |
| 10 | Human adipose stem cells | 48 h |  | 12.4842 |  | Cells combined with platelet rich fibrin | Xu et al. (2016) |
| 11 | Human adipose stem cells | 72 h | 12.4842 |  |  | Cells combined with platelet rich fibrin | Xu et al. (2016) |
| 12 | Human adipose stem cells | 96 h | 12.4842 |  |  | Cells combined with platelet rich fibrin | Xu et al. (2016) |
| 13 | Human adipose stem cells | 120 h | 12.4842 |  |  | Cells combined with platelet rich fibrin | Xu et al. (2016) |
| 14 | Human adipose stem cells | 24 h |  | 12.4842 62.421 124.842 |  | Cells cultured with neural inductive media | Xu et al. (2014) |
| 15 | Human adipose stem cells | 48 h | 12.4842 62.421 124.842 |  |  | Cells cultured with neural inductive media | Xu et al. (2014) |
| 16 | Human adipose stem cells | 72 h | 12.4842 62.421 124.842 |  |  | Cells cultured with neural inductive media | Xu et al. (2014) |
| 17 | Human adipose stem cells | 96 h | 12.4842 62.421 124.842 |  |  | Cells cultured with neural inductive media | Xu et al. (2014) |
| 18 | Human adipose stem cells | 24 h |  | 12.4842 62.421 124.842 |  | Cells cultured with chondrogenic inductive conditioned medium | Xu et al. (2015) |
| 19 | Human adipose stem cells | 48 h |  | 12.4842 62.421 124.842 |  | Cells cultured with chondrogenic inductive conditioned medium | Xu et al. (2015) |
| 20 | Human adipose stem cells | 72 h | 12.4842 62.421 124.842 |  |  | Cells cultured with chondrogenic inductive conditioned medium | Xu et al. (2015) |
| 21 | Human adipose stem cells | 96 h | 12.4842 62.421 124.842 |  |  | Cells cultured with chondrogenic inductive conditioned medium | Xu et al. (2015) |
| 22 | Human periodontal ligament stem cells | 24 h |  | 0.01 0.1 1 10 | 100 | Cells cultured with mineralized  solution | Yin et al. (2015) |
| 23 | Human periodontal ligament stem cells | 48 h | 0.01 0.1 1 10 |  | 100 | Cells cultured with mineralized  solution | Yin et al. (2015) |
| 24 | Human periodontal ligament stem cells | 72 h | 0.01 0.1 1 10 |  | 100 | Cells cultured with mineralized  solution | Yin et al. (2015) |
| 25 | Human periodontal ligament stem cells | 96 h | 0.01 0.1 1 10 |  | 100 | Cells cultured with mineralized  solution | Yin et al. (2015) |
| 26 | Human periodontal ligament stem cells | 120 h | 0.01 0.1 1 10 |  | 100 | Cells cultured with mineralized  solution | Yin et al. (2015) |
| 27 | Human dental pulp stem cells | 72 h | 0.5 2.5 5 10 | 0.1 20 |  |  | Wang et al. (2014) |
| 28 | Human dental pulp cells | 14days | 0.5 2.5 5 10 | 0.1 | 20 |  | Wang et al. (2012) |
| 29 | Rat bone marrow mesenchymal stem cells | 48 h | 1 |  |  |  | Wang et al. (2007) |
| 30 | Rat bone marrow mesenchymal stem cells | 24 h | 0.012484 0.062421 0.12484 0.62421 | 0.00012484 0.0062421 0.0012484 0.0062421 | 1.2484 |  | Fu and Zheng (2013) |
| 31 | Rat bone marrow mesenchymal stem cells | 48 h | 0.012484 0.062421 0.12484 0.62421 | 0.00012484 0.0062421 0.0012484 0.0062421 | 1.2484 |  | Fu and Zheng (2013) |
| 32 | Rat bone marrow mesenchymal stem cells | 72 h | 0.012484 0.062421 0.12484 0.62421 | 0.00012484 0.0062421 0.0012484 0.0062421 | 1.2484 |  | Fu and Zheng (2013) |

# References

Dong, J., Zhu, G., Wang, T.C., and Shi, F.S. (2017). Ginsenoside Rg1 promotes neural differentiation of mouse adipose-derived stem cells via the miRNA-124 signaling pathway. J. Zhejiang Univ. Sci. B 18, 445-448. doi: 10.1631/jzus.B1600355

Fu, X.-K., and Zheng, H.-Z. (2013). The protective effect of ginsenoside Rg1 on rat bone marrow mesenchymal stem cell apoptosis. Chin. J. Clin. 7, 3454-3459. doi: 10.3877/cma.j.issn.1674-0785.2013.08.105

Gu, Y., Zhou, J., Wang, Q., Fan, W., and Yin, G. (2016). Ginsenoside Rg1 promotes osteogenic differentiation of rBMSCs and healing of rat tibial fractures through regulation of GR-dependent BMP-2/SMAD signaling. Sci. Rep. 6, 25282. doi: 10.1038/srep25282

Hu, J., Gu, Y., and Fan, W. (2016). Rg1 protects rat bone marrow stem cells against hydrogen peroxide-induced cell apoptosis through the PI3K/Akt pathway. Mol. Med. Rep. 14, 406-412. doi: 10.3892/mmr.2016.5238

Liu, Y., Yi, L., Wang, L., Chen, L., Chen, X., and Wang, Y. (2016). Ginsenoside Rg1 protects human umbilical cord blood-derived stromal cells against tert-Butyl hydroperoxide-induced apoptosis through Akt-FoxO3a-Bim signaling pathway. Mol. Cell. Biochem. 421, 75-87. doi: 10.1007/s11010-016-2786-y

Wang, L., Wu, X., Lu, X.-Z., Ji, P., Wang, J.-H., Hou, M.-H., et al. (2007). The mechanisms of ginsenosides Rg1 on the proliferation of bone marrow stromal cells. Chin. Pharmacol. Bull. 23, 1480-1484.

Wang, P., Wei, X., Zhang, F., Yang, K., Qu, C., Luo, H., et al. (2014). Ginsenoside Rg1 of Panax ginseng stimulates the proliferation, odontogenic/osteogenic differentiation and gene expression profiles of human dental pulp stem cells. Phytomedicine 21, 177-183. doi: 10.1016/j.phymed.2013.08.021

Wang, P., Wei, X., Zhou, Y., Wang, Y.P., Yang, K., Zhang, F.J., et al. (2012). Effect of ginsenoside Rg1 on proliferation and differentiation of human dental pulp cells *in vitro*. Aust. Dent. J. 57, 157-165. doi: 10.1111/j.1834-7819.2012.01672.x

Xu, F.T., Li, H.M., Yin, Q.S., Cui, S.E., Liu, D.L., Nan, H., et al. (2014). Effect of ginsenoside Rg1 on proliferation and neural phenotype differentiation of human adipose-derived stem cells *in vitro*. Can. J. Physiol. Pharmacol. 92, 467-475. doi: 10.1139/cjpp-2013-0377

Xu, F.T., Li, H.M., Zhao, C.Y., Liang, Z.J., Huang, M.H., Li, Q., et al. (2015). Characterization of chondrogenic gene expression and cartilage phenotype differentiation in human breast adipose-derived stem cells promoted by ginsenoside Rg1 *in vitro*. Cell. Physiol. Biochem. 37, 1890-1902. doi: 10.1159/000438550

Xu, F.T., Liang, Z.J., Li, H.M., Peng, Q.L., Huang, M.H., Li, D.Q., et al. (2016). Ginsenoside Rg1 and platelet-rich fibrin enhance human breast adipose-derived stem cell function for soft tissue regeneration. Oncotarget 7, 35390-35403. doi: 10.18632/oncotarget.9360

Yin, L.H., Cheng, W.X., Qin, Z.S., Sun, K.M., Zhong, M., Wang, J.K., et al. (2015). Effects of ginsenoside Rg-1 on the proliferation and osteogenic differentiation of human periodontal ligament stem cells. Chin. J. Integr. Med. 21, 676-681. doi: 10.1007/s11655-014-1856-9
